# Editorial overview of systematic reviews and meta-analyses on health in the tropics

**DOI:** 10.1186/s41182-019-0174-5

**Published:** 2019-08-06

**Authors:** Nguyen Tien Huy, Heba Elhadad, Nguyen Hai Nam

**Affiliations:** 1 Department of Clinical Product Development, Institute of Tropical Medicine (NEKKEN), School of Tropical Medicine and Global Health, Nagasaki University, Nagasaki, 852-8523 Japan; 2 Online Research Club (http://www.onlineresearchclub.org), Nagasaki, Japan; 3 Department of Parasitology, Medical Research Institute, Alexandria University, Alexandria, Egypt; 4 Department of General Surgery, University of Medicine and Pharmacy, Ho Chi Minh City, Vietnam

The aim of this special issue of *Tropical Medicine and Health* is to draw attention to tropical health problems. Two thirds of the world’s population live in tropical and subtropical regions of Africa, Asia, and Latin America. Many of these people live in absolute poverty and are vulnerable to dozens of diseases. Tropical health problems constitute a huge burden on developing countries, hindering socio-economic development, reducing life quality, impairing physical and cognitive development, causing adverse pregnancy outcomes, and limiting adult productivity within the workforce. This burden is even heavier in poorly resourced communities. In light of this situation, and as stated in Sustainable Development Goals, the World Health Organization is committed to eradicating the epidemics of AIDS, tuberculosis, malaria, and various neglected tropical diseases and combating hepatitis, water-borne diseases, and other communicable diseases by 2030.

Tropical health encompasses a diverse group of issues related to infectious diseases (viral, bacterial, parasitic, and fungal infections), non-communicable diseases, and malnutrition and its consequences. Whereas some of these issues are being properly tackled, others continue to be neglected. On a global scale, Africa, the Eastern Mediterranean region, and Southeast Asia are reported to be the most heavily disease burdened regions, where approximately 50% of all deaths are caused by infectious diseases. Nonetheless, there have also been positive achievements relating to the treatment and control of tropical diseases. Numerous significant studies and interventions during outbreaks of various diseases have been implemented with the aim of controlling and eliminating these diseases. Malaria, dengue, meningitis, and cholera have been effectively maintained in some countries. Also, lymphatic filariasis has been eliminated on some of the Cook Islands, as well as in Niue, the Republic of the Marshall Islands, Tonga, and Vanuatu.

Neglected tropical diseases are often associated with serious disabilities and very high mortality rates, with more than a billion people affected and projections of millions of disability-adjusted life years (DALYs). Such diseases, which include schistosomiasis, food-borne trematode infections, trypanosomiasis, lymphatic filariasis, onchocerciasis, Chagas disease, dracunculiasis, and leishmaniasis, continue to threaten the mental and physical health of people around the world. In the twenty-first century, the magnitude of these problems is anticipated to increase with their spread to more and more countries, which could be attributed to growing populations with diminishing resources, globalization, climatic changes, wars, the collapse of disease control programs in some countries, and the emergence of drug resistance.

Analysis of publication data retrieved from the Scopus database suggests that the number of publications from tropical countries has increased exponentially in the last seven decades, although it is still incomparable to the whole publications worldwide (Fig. 1). Therefore, there is a need for the afflicted countries to play a greater supportive role by encouraging more research on community-based health issues. However, because of limited financial resources available for disease surveillance, the current initiative was proposed to motivate developing countries suffering from these issues to participate in research activities, gather information, and provide the necessary data for clarifying needs and combatting diseases, which seems to be a realistic goal. A systematic review is a type of analysis that entails synthesizing all of the currently available evidence on a specific topic. A meta-analysis, rather than focusing on a single study, entails pooling various studies with analogous designs to determine the overall trend for interpreting an issue, which in the medical field could be disease incidence, overall survival, or the odds ratio for complications. The application of this scientific method reduces bias between studies and increases the reliability and accuracy of the pooled results. We invite systematic reviews alone or together with meta-analyses, as such studies provide robust data with the highest level of evidence. They also provide a low-cost method of summarizing and evaluating the findings of all relevant individual studies including clinical, in vivo, and in vitro studies to improve the health of people in the tropics.

**Fig. 1:**
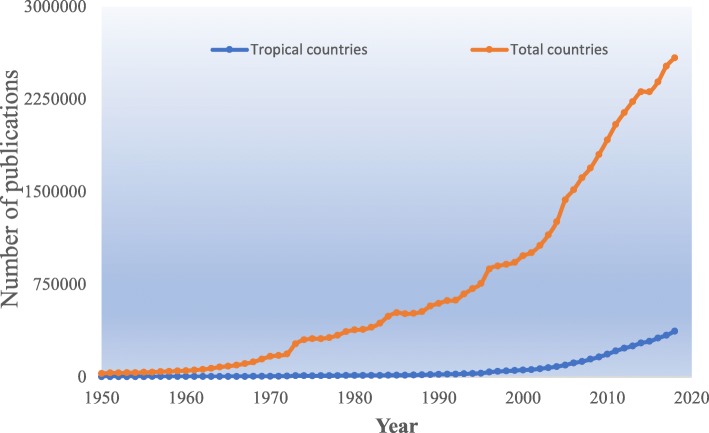
Number of publications with authors from tropical countries compared with the total number of publications

In recognition of a long struggle against tropical diseases, this special issue presents an overview of the current global situation regarding tropical diseases. It reports on progress made in the treatment, control, and elimination of tropical diseases, with a particular methodological focus on systematic reviews and meta-analyses. We hope that these articles will inspire and encourage the involvement of scientists and researchers across the world in the production of scientifically grounded analyses for the purpose of defining disease priorities and formulating measures for managing tropical diseases.

This publication was not supported by any grants.

## Data Availability

None.

